# First person – Jane Khudyakov

**DOI:** 10.1242/bio.039321

**Published:** 2018-11-15

## Abstract

First Person is a series of interviews with the first authors of a selection of papers published in Biology Open, helping researchers promote themselves alongside their papers. Jane Khudyakov is first author on ‘A sample preparation workflow for adipose tissue shotgun proteomics and proteogenomics’, published in BiO. Jane is the PI of her lab at the University of the Pacific, Stockton, USA, investigating comparative physiology & genomics.


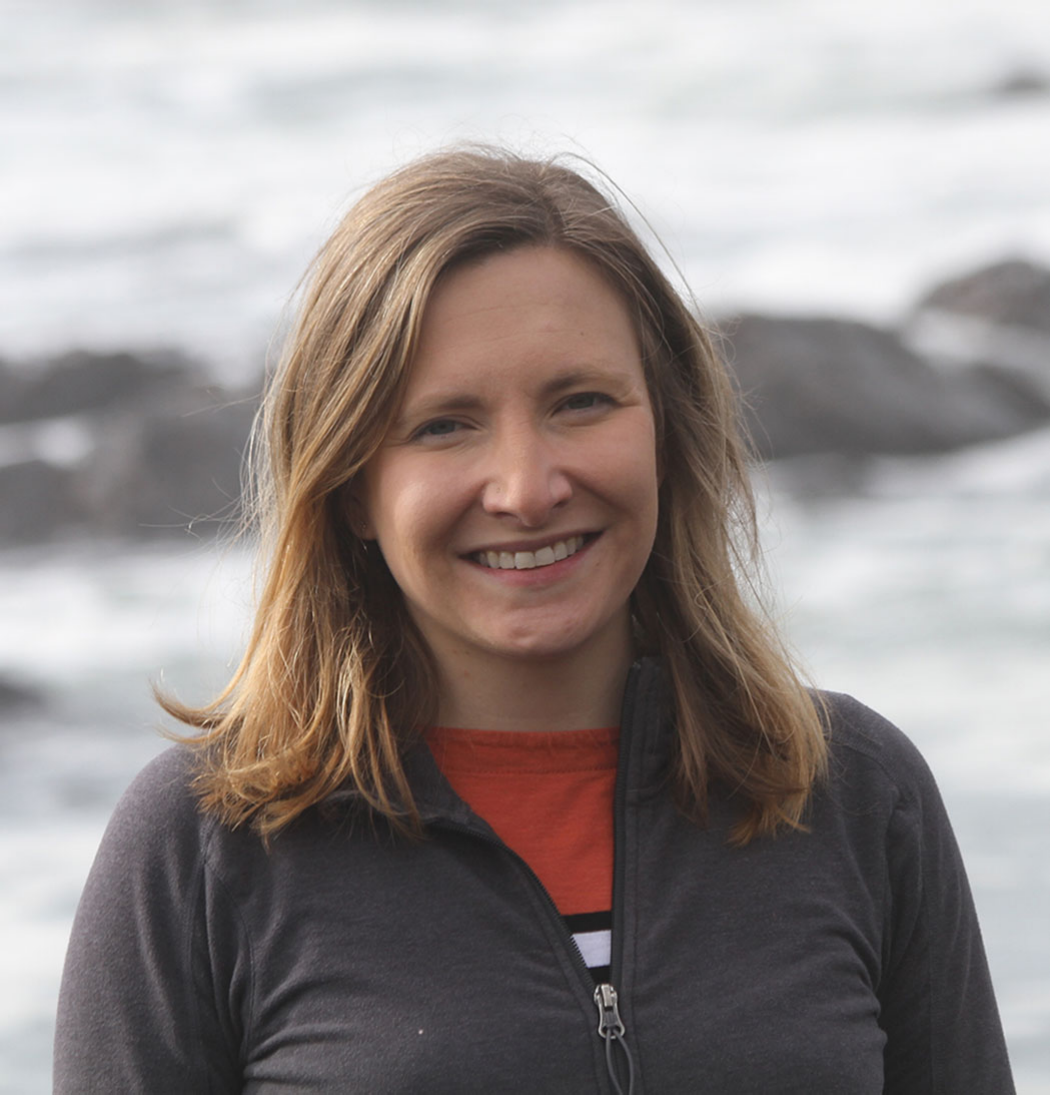


Jane Khudyakov

**What is your scientific background and the general focus of your lab?**

I am a comparative physiologist with a background in molecular, cellular, and developmental biology. My lab studies how animals cope with physiological and environmental challenges such as food and oxygen deprivation and anthropogenic stress. We are currently addressing these topics in marine mammals, which are uniquely adapted to fasting and hypoxia.

**How would you explain the main findings of your paper to non-scientific family and friends?**

Obtaining biological samples from wild marine mammals is extremely difficult. However, measurements of gene and protein activity in tissue samples using modern sequencing technologies can provide large amounts of information about marine mammal health and evolution. One commonly sampled tissue in marine mammals is blubber, a specialized type of subcutaneous fat. Our paper demonstrates a method for simultaneous isolation of high quality RNA and protein from blubber for sequencing applications.

**What are the potential implications of these results for your field of research?**

Our protocol can be used to obtain vast amounts of information (the transcriptome and proteome) from small biopsies of fatty tissue from non-laboratory animals. This can accelerate discoveries in the fields of comparative physiology and evolution.

**What has surprised you the most while conducting your research?**

We have routinely used this protocol (modified phenol-chloroform extraction) to isolate RNA for transcriptomics and have always discarded the ‘contaminating’ proteins. Once we became interested in proteomics, we realized that we could save and purify these proteins for sequencing. While this technique was developed over 30 years ago, there were few reports in the literature on the compatibility of proteins isolated from fatty tissues using this approach with LC-MS/MS.

“Once we became interested in proteomics, we realized that we could save and purify these proteins for sequencing.”

**What, in your opinion, are some of the greatest achievements in your field and how has this influenced your research?**

Application of molecular techniques developed in laboratory species to non-model organisms has yielded incredible insights into animal diversity and evolution. For example, recent studies in hibernating mammals, cephalopods, and flatworms have improved our understanding of cold tolerance, RNA editing and evolution of stem cells in animals. Non-model organisms are treasure troves of information waiting to be discovered, some of which may aid development of new medical treatments and wildlife conservation approaches.

**What changes do you think could improve the professional lives of early-career scientists?**

The biggest improvement would result from increasing public interest and federal investment in scientific research. Funding for research, especially basic science, has been gradually shrinking. This is a major source of stress for junior researchers as their publication records and ability to advance their careers depends on whether they have funding.

**What's next for you?**

Currently, we are working on protein isolation from other tissue types and comparing proteomes responses to prolonged fasting and other stressors in marine mammals.
